# Supplementary material for: Development of an exon skipping therapy for X-linked Alport syndrome with truncating variants in COL4A5
Source: Nat Commun. 2020 Jun 2;11:2777. doi: 10.1038/s41467-020-16605-x (PMC7265383; doi:10.1038/s41467-020-16605-x)
Supplement: Supplementary file 1 — Supplementary Information [file 41467_2020_16605_MOESM1_ESM.pdf]

Supplementary Information

Development of an Exon skipping therapy for X-linked Alport syndrome with truncating variants in *COL4A5*

Tomohiko Yamamura[1], Tomoko Horinouchi[1], Tomomi Adachi[2], Maki Terakawa[2], Yutaka Takaoka[3], Kohei Omachi[4], Minoru Takasato[5], Kiyosumi Takaishi[2], Takao Shoji[6], Yoshiyuki Onishi[6], Yoshito Kanazawa[6], Makoto Koizumi[6], Yasuko Tomono[7], Aki Sugano[3], Akemi Shono[1], Shogo Minamikawa[1], China Nagano[1], Nana Sakakibara[1], Shinya Ishiko[1], Yuya Aoto[1], Misato Kamura[4], Yutaka Harita[8], Kenichiro Miura[9], Shoichiro Kanda[8], Naoya Morisada[1], Rini Rossanti[1], Ming Juan Ye[1], Yoshimi Nozu[1], Masafumi Matsuo[10], Hirofumi Kai[4], Kazumoto Iijima[1] and Kandai Nozu[1*]

1. Department of Pediatrics, Kobe University Graduate School of Medicine, Kobe, Japan
2. Rare Disease Laboratories, Daiichi Sankyo Co., Ltd, Shinagawa, Tokyo, Japan.
3. Division of Medical Informatics and Bioinformatics, Kobe University Hospital, Kobe, Japan
4. Department of Molecular Medicine, Graduate School of Pharmaceutical Sciences, Kumamoto University, Kumamoto, Japan
5. RIKEN Center for Developmental Biology, Kobe, Japan
6. Modality Research Laboratories, Daiichi Sankyo Co., Ltd, Shinagawa, Tokyo, Japan.
7. Division of Molecular Cell Biology, Shigei Medical Research Institute, Okayama, Japan
8. Department of Pediatrics, Graduate School of Medicine, The University of Tokyo, Tokyo, Japan
9. Department of Pediatric Nephrology, Tokyo Women's Medical University, Tokyo, Japan
10. Department of Physical Therapy, Faculty of Rehabilitation, Kobe Gakuin University, Kobe, Japan

Supplementary Table 1 | Nucleotide numbers in each exon of the *COL4A5* gene

Exon 1	81	Exon 18	42	Exon 35	90
Exon 2	60	Exon 19	133	Exon 36	140
Exon 3	90	Exon 20	174	Exon 37	127
Exon 4	45	Exon 21	84	Exon 38	81
Exon 5	45	Exon 22	93	Exon 39	99
Exon 6	63	Exon 23	71	Exon 40	51
Exon 7	54	Exon 24	192	Exon 41	186
Exon 8	27	Exon 25	169	Exon 42	134
Exon 9	81	Exon 26	93	Exon 43	73
Exon 10	63	Exon 27	105	Exon 44	72
Exon 11	36	Exon 28	98	Exon 45	129
Exon 12	42	Exon 29	151	Exon 46	99
Exon 13	93	Exon 30	114	Exon 47	213
Exon 14	54	Exon 31	168	Exon 48	178
Exon 15	57	Exon 32	90	Exon 49	115
Exon 16	45	Exon 33	150	Exon 50	173
Exon 17	54	Exon 34	99	Exon 51	1249

Exons 1–2 and 47–51 belong to the non-collagenous domain.
Numbers in red are a multiple of three.

Supplementary Table 2 | List of all primers in this study

Primer sequences for the mutagenesis in the split nanoluciferase complementation system		
c.1350_1351delAT	Forward:	ATGAGATGGGCTCGAGCGGTGGTGGCGGGA
	Reverse:	TCGAGCCCATCTCATCACTAGGAGGAATGT
c.1411C>T	Forward:	ATGAGATGGGCTCGAGCGGTGGTGGCGGGA
	Reverse:	TCGAGCCCATCTCATCACTAGGAGGAATGT
c.1340_1423del84bp	Forward:	TCCTCCTAGTGACAAAGGTGACACTTGCTTC
	Reverse:	TTGTCACTAGGAGGAATGTGAGGGCCAGCAG
Primer sequences for the RT-PCR in the In vitro exon skipping efficiency evaluation by ASO		
COL4A5 cDNA primer	Forward:	TTGCCTGGAGAAAAAGGAGA
	Reverse:	TTCTGTCCAGGGAAACCAAG
Primer sequences for the RT-PCR in the In vivo (mouse) exon skipping evaluation by ASO		
Col4a5 cDNA primer	Forward:	GGGGCCAGAAAGGTGATGAA
	Reverse:	AAAGCCTGGAGCACCTGGAG

Supplementary Table 3 | Clinical and laboratory data about the mice studied

		Weight (g)				
		Whole body	Kidney	Heart	Liver	Pancreas
Average	Wild-type (n = 3)	30.1	0.34	0.13	1.48	0.07
	Vehicle (n = 6)	29.5	0.31	0.16	1.22	0.08
	ASO (n = 5)	28.70	0.34	0.14	1.55	0.08
SE	Wild-type	2.20	0.02	0.00	0.03	0.00
	Vehicle	1.02	0.02	0.00	0.00	0.00
	ASO	0.58	0.01	0.00	0.00	0.00

		Serum laboratory data		
		Albumin (g/dL)	BUN (mg/dL)	Creatinine (mg/dL)
Average	Wild-type (n = 3)	2.77	33.00	0.10
	Vehicle (n = 6)	2.42	83.67	0.35
	ASO (n = 5)	2.36	34.20	0.12
SE	Wild-type	0.07	1.00	0.00
	Vehicle	0.04	8.32	0.05
	ASO	0.07	3.43	0.02

SE: standard errors

Source data are provided as a Source Data file.

A

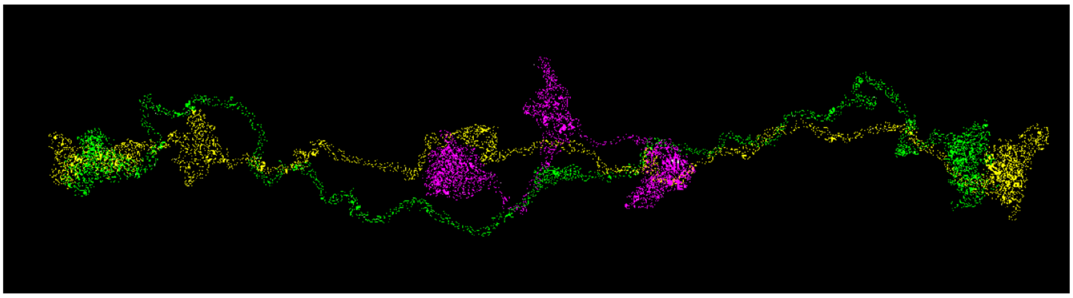

B

Collagen	Wild Type	Q471X	Δexon21
Total Energy (kJ/mol)	84044.9	43524.4	74988.2

Supplementary Fig. 1 | 3D structure of collagen IV. a, 3D structure of collagen IV: Green, wild-type; yellow, p.D448_G475del84bp mutant; Magenta, p.Gln471* mutant. **b**, Total energy of each structure. The energy value for Δexon21 is closer to the wild-type value. Source data are provided as a Source Data file.

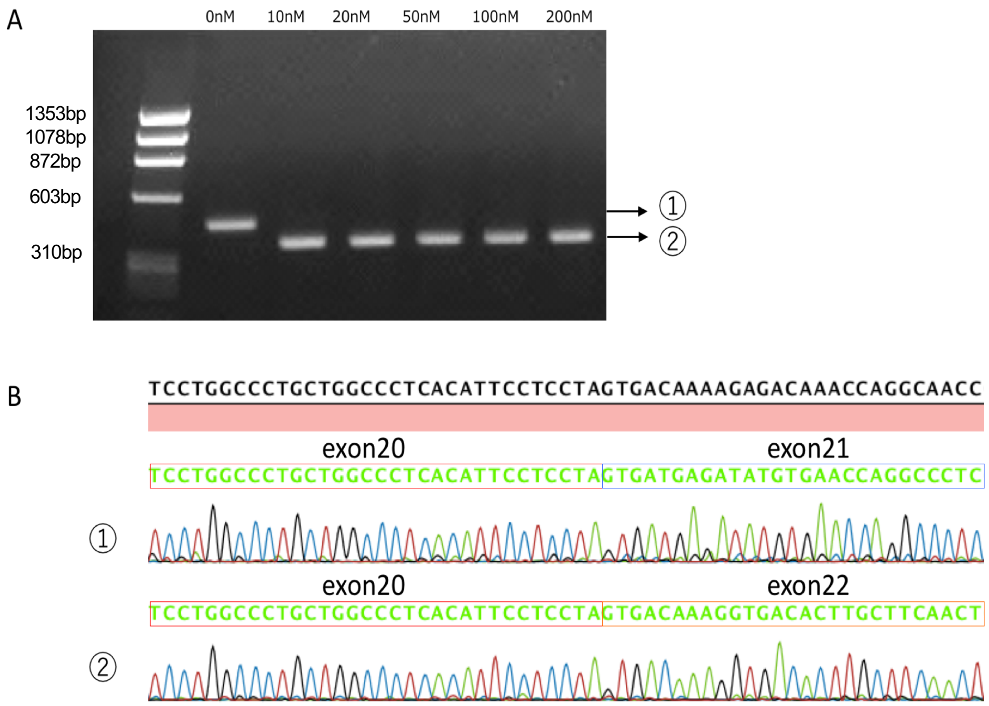

Supplementary Fig. 2 | In vitro exon skipping efficiency evaluation by an antisense oligonucleotide (ASO). Patient's urine derived cultured cells (p.Gln471* in exon 21) were transfected with an ASO that targeted exon 21 (84 bp) skipping. **a**, even the lowest dose of 10 nM caused complete exon 21 skipping. **b**, The sequencing results showed that the larger band included exons 20 to 22, whereas the sequencing results of the smaller band showed that exon 20 was directly connected to exon 22. RT-PCR was conducted twice and gave identical results.

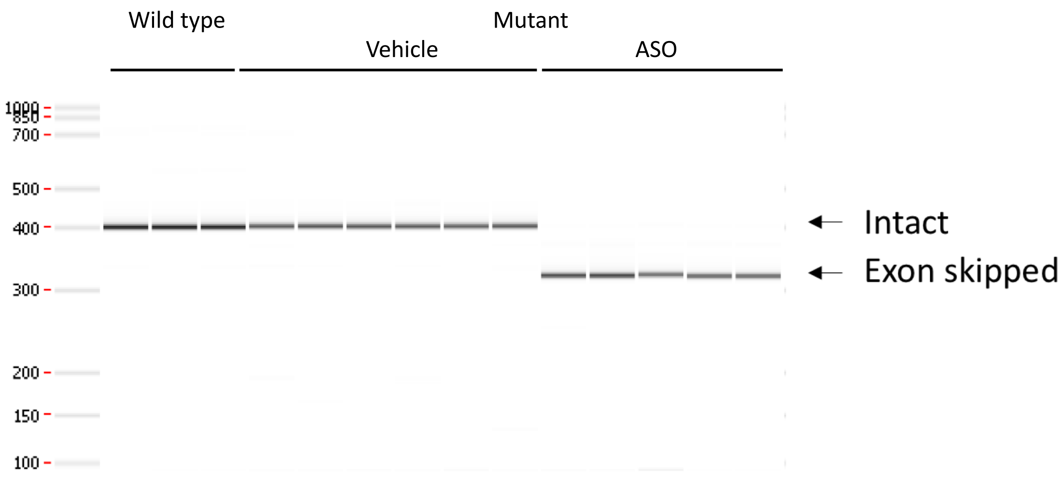

Supplementary Fig. 3 | RT-PCR results for treated mice kidneys. Wild-type and vehicle treated mice had larger PCR products with exon 21 present, whereas the antisense oligonucleotide (ASO) treated mice gave rise to a band that showed that exon 21 had been skipped, that is, shorter oligonucleotide product. Primer sequences are shown in Supplementary Table 2. RT-PCR was conducted once for all samples and samples in the treatment group gave an identical exon-skipped pattern as shown in this figure.

WT (saline)

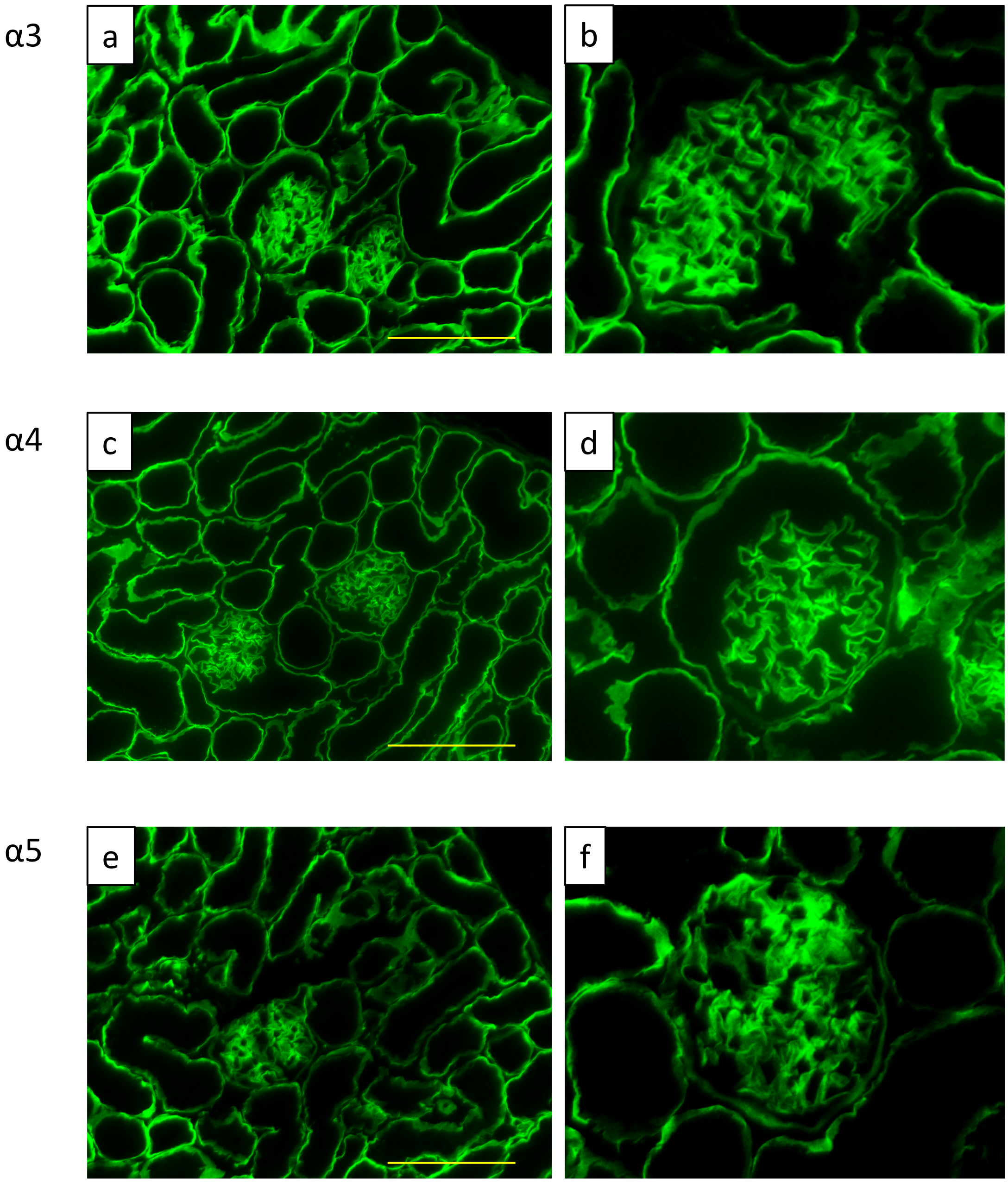

MUT (saline)

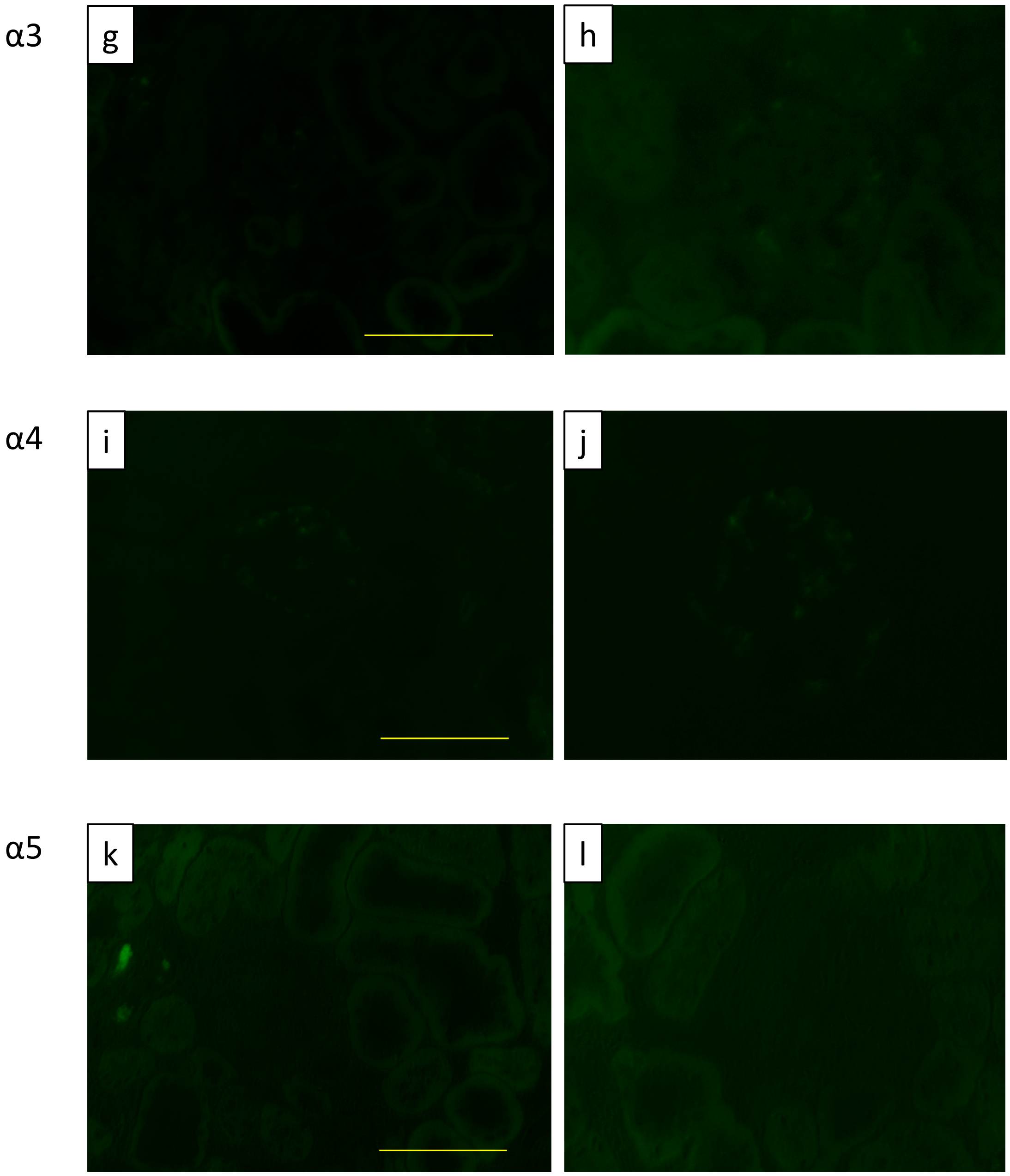

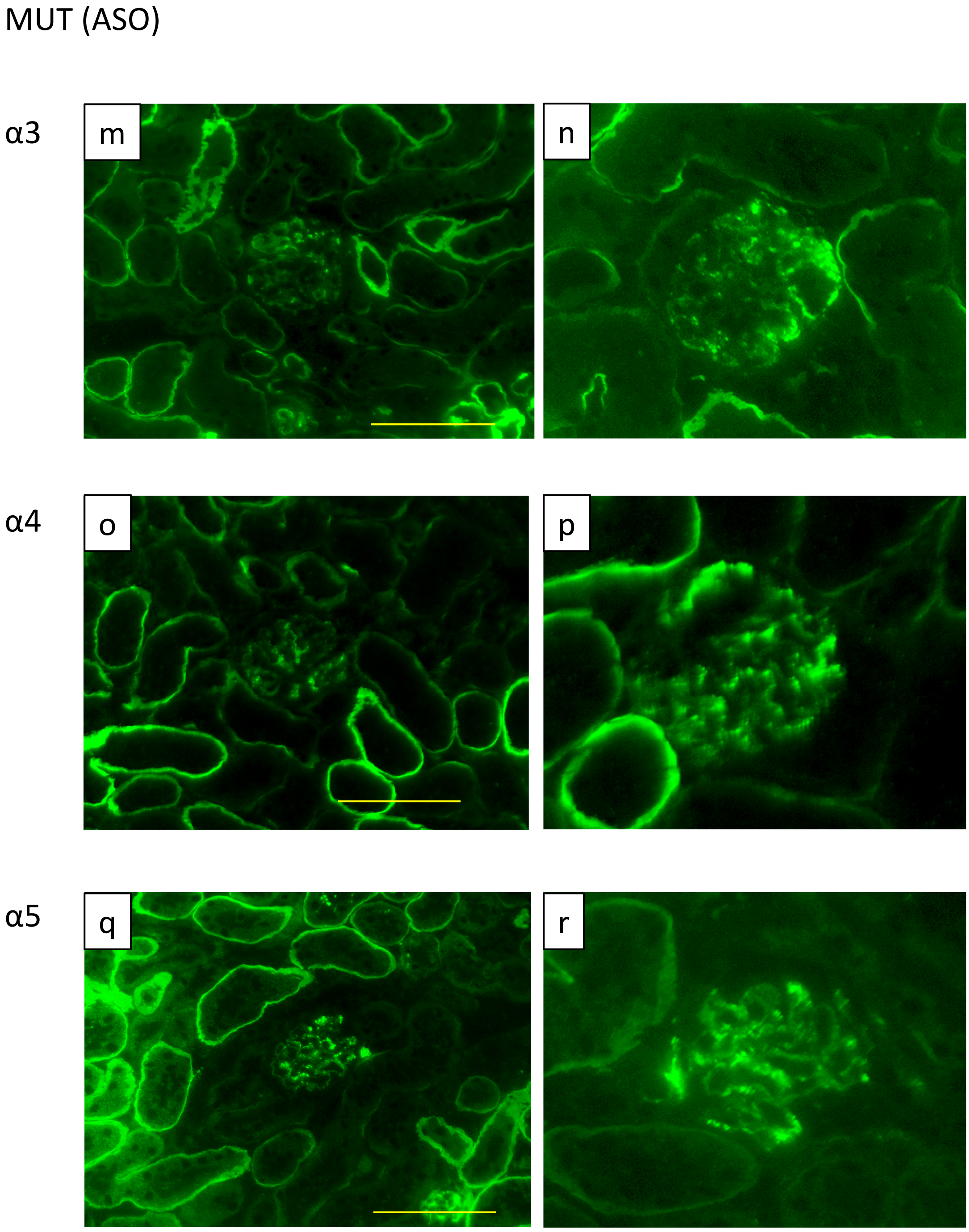

Supplementary Fig. 4 |Type IV collagen α3/α4/α5 chain staining at twenty-one weeks of age. Green: Type IV collagen α3/α4/α5 chain. Type IV collagen α3/α4/α5 chain were completely negative in the vehicle group (g-l), but they were expressed on the glomerular basement membrane (GBM) in ASO-treated group (m-r). Scale bars are 100 μm. Staining was conducted by two different researchers from different institutes and gave identical expression patterns.

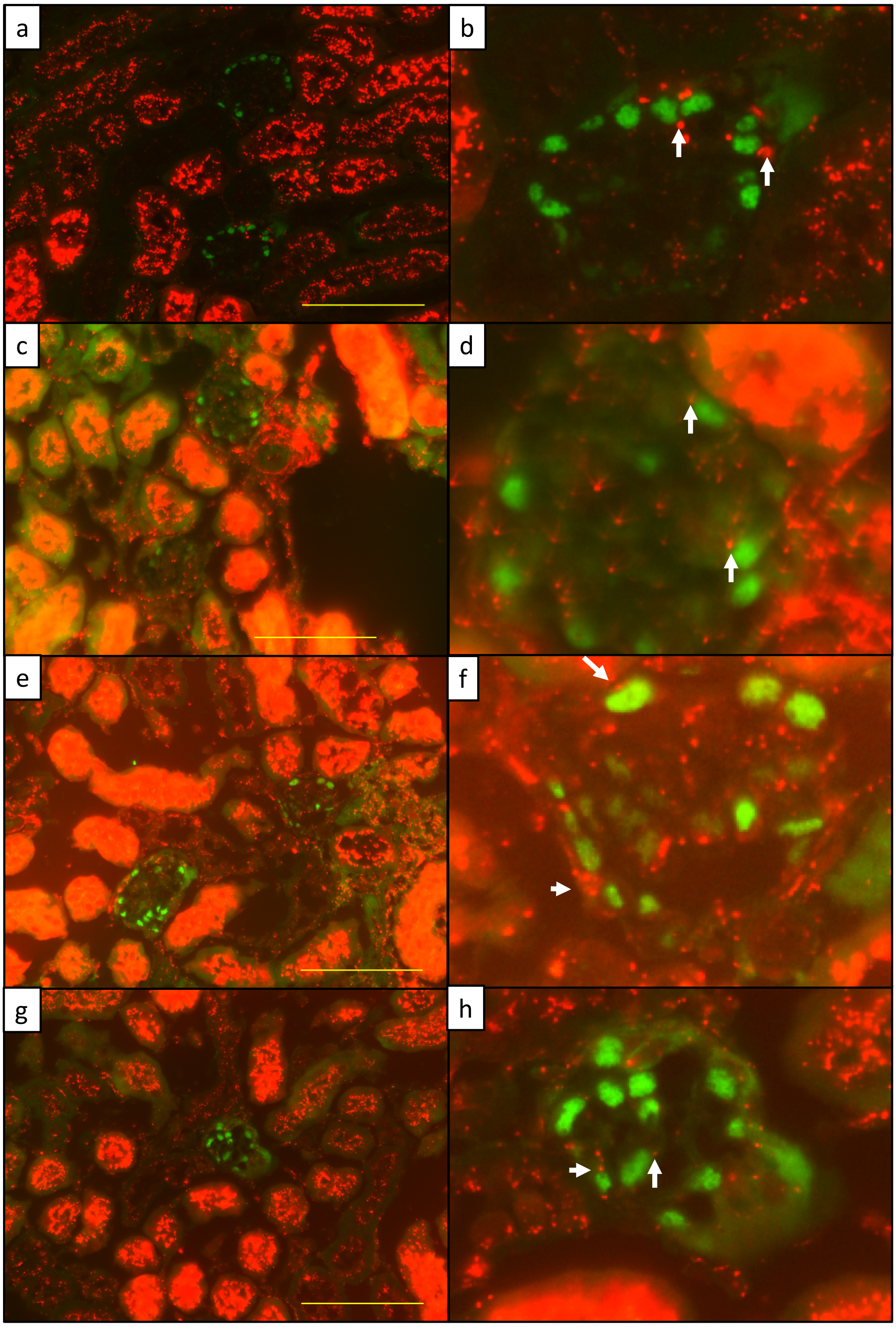
a
b
c
d
e
f
g
h

Supplementary Fig. 5 | ASO-ENA injection into healthy mice. Green: WT1 protein. Red: ASO-ENA tagged by Cyanine 3. The uptake of ASO-ENA by podocytes (arrows) and tubular epithelial cells (arrow heads) is clearly observed in every specimen. **a, b,** Wild-type mouse sacrificed 24 hours after administration. **b, c,** Mutant mouse sacrificed 24 hours after administration. **e, f,** Wild-type mouse sacrificed two weeks after administration. **g, h,** Mutant mouse sacrificed two weeks after administration. Scale bars are 100 μm. Staining was conducted twice and both yielded the same staining pattern.
