# Supplementary material for: Development of an exon skipping therapy for X-linked Alport syndrome with truncating variants in COL4A5
Source: Nat Commun. 2020 Jun 2;11:2777. doi: 10.1038/s41467-020-16605-x (PMC7265383; doi:10.1038/s41467-020-16605-x)
Supplement: Supplementary file 3 — Reporting Summary [file 41467_2020_16605_MOESM3_ESM.pdf]

nature research

Corresponding author(s): Kandai Nozu

Last updated by author(s): Mar 19, 2020

Reporting Summary

Nature Research wishes to improve the reproducibility of the work that we publish. This form provides structure for consistency and transparency in reporting. For further information on Nature Research policies, see Authors & Referees and the Editorial Policy Checklist .

Statistics

For all statistical analyses, confirm that the following items are present in the figure legend, table legend, main text, or Methods section.

n/a	Confirmed	
☐	☒	The exact sample size (*n*) for each experimental group/condition, given as a discrete number and unit of measurement
☐	☒	A statement on whether measurements were taken from distinct samples or whether the same sample was measured repeatedly
☐	☒	The statistical test(s) used AND whether they are one- or two-sided<br>*Only common tests should be described solely by name; describe more complex techniques in the Methods section.*
☒	☐	A description of all covariates tested
☒	☐	A description of any assumptions or corrections, such as tests of normality and adjustment for multiple comparisons
☐	☒	A full description of the statistical parameters including central tendency (e.g. means) or other basic estimates (e.g. regression coefficient) AND variation (e.g. standard deviation) or associated estimates of uncertainty (e.g. confidence intervals)
☐	☒	For null hypothesis testing, the test statistic (e.g. *F*, *t*, *r*) with confidence intervals, effect sizes, degrees of freedom and *P* value noted<br>*Give P values as exact values whenever suitable.*
☒	☐	For Bayesian analysis, information on the choice of priors and Markov chain Monte Carlo settings
☒	☐	For hierarchical and complex designs, identification of the appropriate level for tests and full reporting of outcomes
☒	☐	Estimates of effect sizes (e.g. Cohen's *d*, Pearson's *r*), indicating how they were calculated

Our web collection on statistics for biologists contains articles on many of the points above.

Software and code

Policy information about availability of computer code

Data collection	No software was used.
Data analysis	JMP13, Excel

For manuscripts utilizing custom algorithms or software that are central to the research but not yet described in published literature, software must be made available to editors/reviewers. We strongly encourage code deposition in a community repository (e.g. GitHub). See the Nature Research guidelines for submitting code & software for further information.

Data

Policy information about availability of data

All manuscripts must include a data availability statement. This statement should provide the following information, where applicable:
- Accession codes, unique identifiers, or web links for publicly available datasets
- A list of figures that have associated raw data
- A description of any restrictions on data availability

The data that support the findings of this study are available from the corresponding author upon reasonable request. The source data underlying Figs. 1a, b, 5a, 5b, 5c, 5d, 5e, and Supplementary Figs. 1 Supplementary Tables 3 are provided as a Source Data file.

Field-specific reporting

Please select the one below that is the best fit for your research. If you are not sure, read the appropriate sections before making your selection.

☒ Life sciences ☐ Behavioural & social sciences ☐ Ecological, evolutionary & environmental sciences

Life sciences study design

All studies must disclose on these points even when the disclosure is negative.

Sample size	No statistical tests were used to determine sample size. For Split luciferase-based trimer formation of the α345(IV) proteins assay, we perform 6 independent experiment repeats to allow statistical analysis. For 3-D structure analysis of collagen IV, we perform experiment only one time because it is a computer analysis. For mice study, based on previous report in this model mouse (PMID: 30582011), it was determined that numbers of each group with 5 or 6 is sufficient to evaluate the therapeutic effect. Therefore, We randomly divided litter mates into approximately half (5 and 6).
Data exclusions	No data were excluded from the analyses.
Replication	Figure 1, Split luciferase-based trimer formation of the α345(IV) proteins assay; Each assay was conducted 6 times.<br>Figure 2, 3-D structure analysis of collagen IV; No replication was conducted.<br>Figure 3, Supplementary Figure 4; alpha 3/4/5 IF was conducted by two different researchers from different institute (Dr. Yamamura for Fig 3 and Dr. Tomono for Sup Fig 4) and had completely the same expression pattern.<br>Figure 4, 5; We conducted not only the mice study up to 21 weeks of age for clinicopathological evaluation, but also the additional study to prove In addition substantial renal rescue effect of exon 21 skipping by long term mice study. As a result, we showed significant therapeutic effect on both study. Therefore, it is determined that there is a clear reproducibility.<br>Supplementary Figure 2; RT-PCR was conducted twice and both showed same pattern.<br>Supplementary Figure 3; RT-PCR was conducted for all sample one time and samples in each treatment group showed completely same pattern as shown in Figure.<br>Supplementary Figure 5; Staining was conducted twice and both showed the same staining pattern.
Randomization	In the mice study, each mouse (litter mate) was randomly assigned as vehicle or ASO treated group.
Blinding	The study was not blinded because it was evaluated by objective indicators such as biochemical data and substantial survival rate. But this study was conducted by external company (Axcelead Drug Discovery Partners) and evaluated the efficacy by them.

Reporting for specific materials, systems and methods

We require information from authors about some types of materials, experimental systems and methods used in many studies. Here, indicate whether each material, system or method listed is relevant to your study. If you are not sure if a list item applies to your research, read the appropriate section before selecting a response.

Materials & experimental systems

n/a	Involved in the study	
☐	☒	Antibodies
☐	☒	Eukaryotic cell lines
☒	☐	Palaeontology
☐	☒	Animals and other organisms
☐	☒	Human research participants
☒	☐	Clinical data

Methods

n/a	Involved in the study	
☒	☐	ChIP-seq
☒	☐	Flow cytometry
☒	☐	MRI-based neuroimaging

Antibodies

Antibodies used	The following antibodies were used in this study<br>1. Rabbit Anti-Wilms Tumor Protein antibody for immunofluorescence staining analysis, abcam, ab89901, Monoclonal (Clone CAN-R9(IHC)-56-2) Lot: GR177328-39.<br>2. Rat Anti-Collagen IV α3(IV) Chain antibody for immunofluorescence staining analysis, Shigei Medical Research Institute, not for sale, Monoclonal (clone 129).<br>3. Rat Anti-Collagen IV α4(IV) Chain antibody for immunofluorescence staining analysis, Shigei Medical Research Institute, not for sale, Monoclonal (clone b42).<br>4. Rat Anti-Collagen IV α5(IV) Chain antibody for immunofluorescence staining analysis, Shigei Medical Research Institute, SGE-C-453, Monoclonal (clone H53).<br>5. Goat anti-Rat IgG (H+L) Cross-Adsorbed Secondary Antibody, Alexa Fluor 488, Invitrogen, Polyclonal<br>6. Goat anti-Rabbit IgG (H+L) Cross-Adsorbed Secondary Antibody, Alexa Fluor 546, Invitrogen, Polyclonal
Validation	All antibody in our study have been validated. Detailed information could be found on the manufactures' web site or relevant citations listed below.<br>1. Wilms Tumor protein (ab89901), https://www.abcam.co.jp/wilms-tumor-protein-antibody-can-r9ihc-56-2-ab89901-protocols.html<br>2. Collagen IV α3(IV) Chain (clone 129) have been validated in previous report, https://www.ncbi.nlm.nih.gov/pubmed/?

term=High+nephritogenicity+of+monoclonal+antibodies+belonging+to+IgG2a+and+IgG2b+subclasses+in+rat+anti-GBM+nephritis
3. Collagen IV α4(IV) Chain (clone b42) have been validated in previous report, https://www.ncbi.nlm.nih.gov/pubmed/?term=High+nephritogenicity+of+monoclonal+antibodies+belonging+to+IgG2a+and+IgG2b+subclasses+in+rat+anti-GBM+nephritis
4. Collagen IV α5(IV) Chain (clone H53), https://www.cosmobiousa.com/products/anti-collagen-4-alpha-5-iv-mab-clone-h53-fluorescein-labeled
5. Goat anti-Rat IgG (H+L) Cross-Adsorbed Secondary Antibody, https://www.thermofisher.com/antibody/product/Goat-anti-Rat-IgG-H-L-Cross-Adsorbed-Secondary-Antibody-Polyclonal/A-11006
6. Goat anti-Rabbit IgG (H+L) Cross-Adsorbed Secondary Antibody, https://www.thermofisher.com/antibody/product/Goat-anti-Rabbit-IgG-H-L-Cross-Adsorbed-Secondary-Antibody-Polyclonal/A-11010

Eukaryotic cell lines

Policy information about cell lines

Cell line source(s)	HEK293T cells (RCB2202) and Patient's Urine derived cells were used for this study.
Authentication	None of the cell lines were authenticated.
Mycoplasma contamination	The cell lines are not tested for Mycoplasma contamination.
Commonly misidentified lines (See ICLAC register)	None of the cell lines used are listed in the ICLAC database.

Animals and other organisms

Policy information about studies involving animals; ARRIVE guidelines recommended for reporting animal research

Laboratory animals	Col4a5 mutant male mouse model with c.1411C>T (p.Arg471*) with background of 57BL/6J was used for this study.<br>For In vivo exon skipping efficiency evaluation by ASO using a mouse model. We started the treatment twice a week from four to 6 weeks of age and once a week from 7 to 20 weeks of age. We sacrificed mice at 21 weeks of age and harvested samples.<br>For In vivo drug delivery evaluation of ASO using a mouse model. we administrated a single dose of ASO-ENA at 6 weeks of age. We sacrificed mice 24 h or 2 weeks.<br>All mice were kept in a regular 12 hours light, 12 hours dark cycle under specific pathogen free condition at 25 degree Celsius.
Wild animals	no wild animals were used in this study.
Field-collected samples	no field-collected samples were used in this study.
Ethics oversight	All animal experiments were approved by the Committee on Animal Experimentation at Daiichi Sankyo Co., Ltd and Axcelead Drug Discovery Partners, Inc. Japan. Animals were treated in accordance with the Guide for Animal Experimentation.

Note that full information on the approval of the study protocol must also be provided in the manuscript.

Human research participants

Policy information about studies involving human research participants

Population characteristics	Urine derived cell was collected from a nine-year-old boy with X-linked Alport syndrome caused by the nonsense mutation in COL4A5 gene.
Recruitment	We recruited this participant because he had a truncating variant in COL4A5 gene and can be potential target of the future therapy.
Ethics oversight	All procedures were reviewed and approved by the Institutional Review Board of Kobe University School of Medicine (No. 301 and 1451).

Note that full information on the approval of the study protocol must also be provided in the manuscript.
